# Improved Neural Processing Efficiency in a Chronic Aphasia Patient Following Melodic Intonation Therapy: A Neuropsychological and Functional MRI Study

**DOI:** 10.3389/fneur.2016.00148

**Published:** 2016-09-19

**Authors:** Ken-ichi Tabei, Masayuki Satoh, Chizuru Nakano, Ai Ito, Yasuo Shimoji, Hirotaka Kida, Hajime Sakuma, Hidekazu Tomimoto

**Affiliations:** ^1^Department of Dementia Prevention and Therapeutics, Graduate School of Medicine, Mie University, Mie, Japan; ^2^Department of Neurology, Graduate School of Medicine, Mie University, Mie, Japan; ^3^Department of Rehabilitation, Suzuka Central General Hospital, Mie, Japan; ^4^Department of Radiology, Graduate School of Medicine, Mie University, Mie, Japan

**Keywords:** aphasia, neural efficiency theory, functional magnetic resonance imaging, Western Aphasia Battery, prosody, Japanese version of MIT

## Abstract

Melodic intonation therapy (MIT) is a treatment program for the rehabilitation of aphasic patients with speech production disorders. We report a case of severe chronic non-fluent aphasia unresponsive to several years of conventional therapy that showed a marked improvement following intensive 9-day training on the Japanese version of MIT (MIT-J). The purpose of this study was to verify the efficacy of MIT-J by functional assessment and examine associated changes in neural processing by functional magnetic resonance imaging. MIT improved language output and auditory comprehension, and decreased the response time for picture naming. Following MIT-J, an area of the right hemisphere was less activated on correct naming trials than compared with before training but similarly activated on incorrect trials. These results suggest that the aphasic symptoms of our patient were improved by increased neural processing efficiency and a concomitant decrease in cognitive load.

## Introduction

It is well known in clinical practice that patients with severe non-fluent aphasia can sing well without prompting (1–3). Previous studies have suggested that verbal production, whether sung or spoken, originates from the same neural processes. In patients with aphasia, there is a large overlap between the neural activation patterns elicited by disyllabic words or phrases when either sung or spoken (3, 4). In contrast, in normal individuals, neural activity is higher during singing than speaking, and the pattern of neural activation is strong in the anterior to middle portions of the superior temporal gyrus, and markedly stronger in the right than the left hemisphere during singing (5). Because of these findings, Ozdemir et al. (5) proposed two possible routes for the articulation of words: (i) a purely language-based route in the left hemisphere and (ii) a singing-based or melodically intoned route that is either right hemispheric or bihemispheric.

Different rehabilitative approaches for aphasia are available (6, 7) based on psycholinguistic (8, 9), cognitive (10), and psychosocial or pragmatic (11–13). Melodic intonation therapy (MIT) is a hierarchically structured treatment program identified by the American Academy of Neurology as an effective form of output-focused language therapy (14, 15). The original version of MIT has been adapted for various clinical populations, including non-English linguistic populations such as Romanian (16), Persian (17), Italian (18), and Japanese (19), with comparable clinical results. MIT is based on the assumption that the stress, intonation, and melodic patterns of language output (prosody) are controlled primarily by the right hemisphere and is thus preserved in individuals with aphasia because of left hemisphere damage (20–24). MIT is intended to engage the right hemisphere given its dominant role in processing spectral information, global features of music, and prosody, whereas left-hand tapping may engage a right-hemisphere sensorimotor network that controls both hand and mouth movements (25–27).

Although MIT may induce functional and structural changes in the right hemisphere (28–32), it is not yet clear whether it is stress, intonation, melodic pattern, or some combination of these factors that aids in speech production (33, 34). Some recent neuroimaging studies support right hemisphere involvement in MIT (31, 32), whereas others do not (35). Alternatively, some studies have concluded that MIT promotes left perilesional activation (36, 37). The different functional imaging techniques used across studies may account, at least in part, for these inconsistencies (38). Thus, the neural processes that participate in MIT and the changes underlying clinical improvement remain unclear.

In this study, we report a patient with severe non-fluent aphasia who received the Japanese version of MIT (MIT-J) for nine consecutive days, 3 years after the onset of aphasia. The purpose of this study was to verify the efficacy of MIT-J and to identify the underlying neural processes that are altered by MIT using functional MRI (fMRI).

## Case History

**Figure 1:**
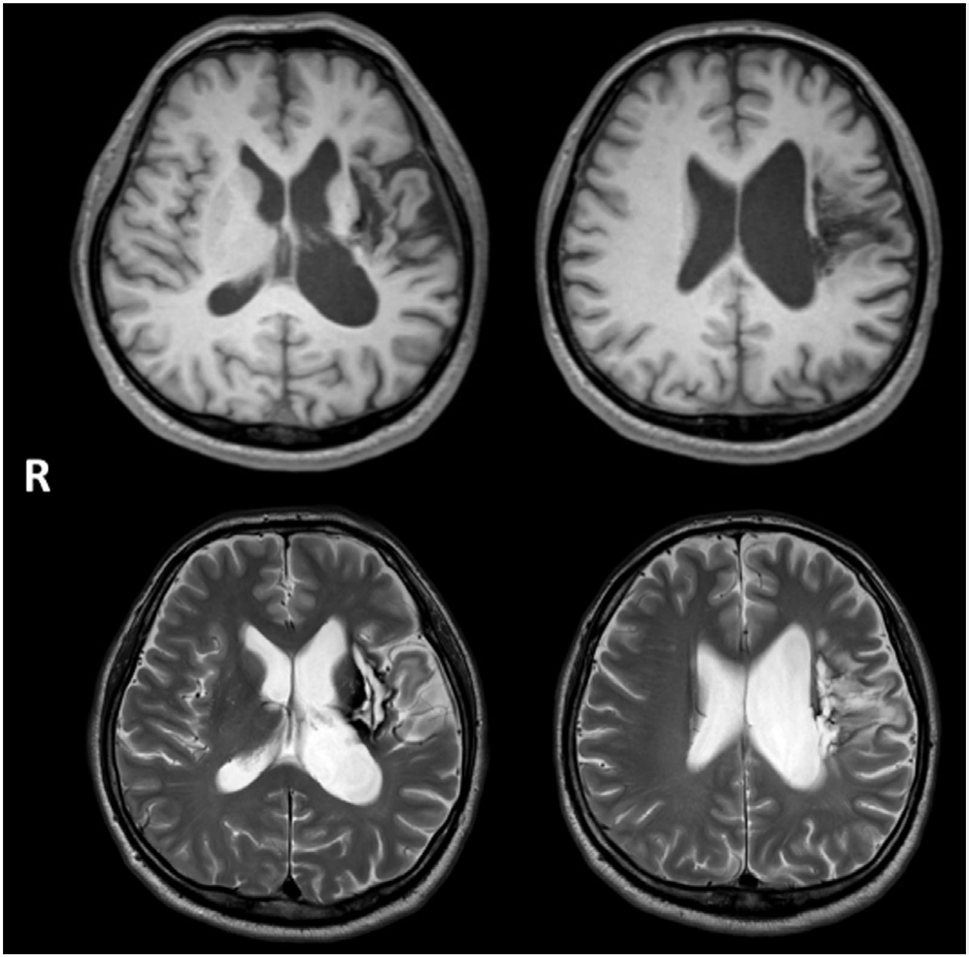
**Axial MRI images showing the lesion resulting from a left putaminal hemorrhage, which had spread to thalamus and subcortical regions of the frontotemporal lobe**. Upper and lower images show T1- and T2-weighted images, respectively.

A 48-year-old right-handed male developed right hemiparesis and aphasia associated with left putaminal hemorrhage. Axial MRI images in Figure 1 showing the lesion resulting from a left putaminal hemorrhage, which had spread to thalamus and subcortical regions of the frontotemporal lobe. He received 2 months of rehabilitation during hospitalization. Although linguistic comprehension was relatively well preserved, he could utter words only with great effort and hesitation, symptoms consistent with motor aphasia. Therefore, for 2 years after the event, he received weekly speech training from a speech therapist (Yasuo Shimoji), but his symptoms remained unchanged. Three years after onset, he was admitted in our hospital for intensive training using MIT-J.

Neurological examination revealed right hemiparesis and hypoesthesia on the right side of his body, but there was no evidence of unilateral neglect, apraxia, dyscalculia, or visual discrimination difficulties. No hearing deficit was found. He was able to eat with chopsticks using his left hand and use the toilet unaided. He also walked independently with a lower extremity orthosis. However, assistance was need for changing clothes and getting into the bath. Neuropsychological assessment results included a normal score on the Japanese version of Raven’s Colored Progressive Matrices (32/36) (39). Memory function was also normal as evaluated using the Japanese version of the Benton Visual Retention Test (BVRT) (40): Test A correct score = 10 (mean = 7), error score = 2; Test B correct score = 6 (mean = 6), error score = 6. He could also correctly copy the figure of a cube; results of the Trail Making Test (TMT)-A and -B (41) were as follows: A = 137 s (normal population, mean ± SD; 119.6 ± 44.5) and B = 332 s (mean ± SD; 158.1 ± 53.6). The prolongation of TMT-B was attributable to aphasia, so we regarded frontal function of the patient as normal. Brain MRI revealed a lesion from the left putaminal hemorrhage, which had spread to thalamus and subcortical regions of the frontotemporal lobe (Figure 1).

All procedures followed the Clinical Study Guidelines of the Ethics Committee of Mie University Hospital, Japan and were approved by the internal review board. A complete description of all procedures was provided to the patient.

## Methods

### Japanese Version of MIT

The procedure (level and step) of MIT-J was identical to the original MIT, consisting of four levels (22). Level I is the humming the intonation of the target word. The tasks in Level II move from requiring the subject to tap the rhythm of the clinician’s intoned utterances to repetition of a target sentence. At Level III, difficulty is increased by progressively decreased participation of the clinician and by first introducing, and finally requiring, the subject to give appropriately intoned responses to intoned questions from the clinician regarding elements of presented sentences. At Level IV, the latency between stimulus and response is increased to produce decay of repetition skill and to increase efficiency of retrieval. Transition back to speech prosody is facilitated by a technique called “speech-song,” in which the melodic line remains the same as the intoned sentence of the preceding step, except that the constant pitch of intoned words is replaced by the variable pitch of speech. During all levels, tapping of the left hand is simultaneously used.

Japanese version of MIT as developed by Seki and Sugishita (19) is based on the original, but modified for the unique grammatical and phonological characteristics of Japanese (Figure S1 in Supplementary Material). The phonological unit in the Japanese language is a mora (42), a temporal unit that divides words into almost isochronous segments (43). Although a syllable and a mora are distinct conceptual units, many syllables in Japanese have just one mora (44). Thus, there are two major differences in MIT-J compared with the original MIT. First, the MIT-J uses two pitches, high and low, whereas the original MIT has several pitches. Second, in MIT-J, there are two moras in a single beat of left-hand tapping. These modifications may make MIT-J easier and more effective for broader aphasic symptoms than the original. In the present case, a speech therapist (Chizuru Nakano), who is also a licensed music therapist of Japan, administered the MIT-J for 45 min/day for nine consecutive days. The target words were chosen based on the severity of aphasia and the necessities in the daily life of the patient. The patient completed all other interventions during the hospitalization period.

### Assessment of Linguistic Function

We used two tests to assess linguistic function: the Japanese version of the Western Aphasia Battery (WAB) (45) and the naming of 90 words. The WAB assesses six aspects of language function: spontaneous speech, auditory comprehension, repetition, naming, reading, and writing. The aphasia quotient (AQ) is used to express the overall linguistic function of the patient. For the naming of 90 words, we chose words with high “imageability” (80%), where “imageability” expresses the ease of evoking various mental images of the word (46). We presented the figures of these words, which were not used in the MIT-J intervention, on the screen of a personal computer using Cards for Speech Training (ActCard, Escor Co. Ltd.). In addition to the correct response score, we measured the response time (RT) from presentation of the figure to naming using sound analyzing software (Audacity 2.0.5., Audacity Team). These assessments were performed before and after MIT-J.

### Functional MRI Assessment

To examine changes in brain activity associated with MIT-J, we performed fMRI during the word-naming task before and after completion of the intervention. The stimuli were 36 of the 90 figures used for assessment of naming. Eighteen stimuli were correct, and the others were incorrect in the results. Stimuli were displayed using magnetic resonance compatible goggles (CinemaVision, Resonance Technology Inc., CA, USA), controlled using E-Prime software (Psychology Software Tools, Inc., PA, USA) on a personal computer. Following the presentation of a fixation point (cross) for 2 s, a figure was presented for 6 s, and the patient was required to name it as quickly as possible. Thirty-six figures and 12 blanks as baseline were presented 3 times, so the total number of performance trials was 144. Using a noise-canceling microphone set near the patient’s mouth, we were able to monitor his performance through a speaker in the operation room.

### fMRI Measurements

All images were acquired using a 3.0-T MR scanner (Achieva Quasar dual 3.0-Tesla, Koninklijke Philips Electronics). Functional images were obtained using a T2*-weighted gradient-echo echo planar imaging sequence [repetition time (TR) = 3,000 ms, echo time (TE) = 35 ms, flip angle = 90°, slice thickness = 5 mm, gapless, field of view (FOV) = 240 mm, 96 × 96 matrix]. The voxel size was 2.5 mm × 2.5 mm × 5 mm. In addition, a T1-weighted anatomical image was obtained (TR = 7.6 ms, TE = 3.6 ms, flip angle = 8°, slice thickness = 0.7 mm, FOV = 250 mm × 250 mm, in-plane resolution = 1.04 mm × 1.04 mm).

### fMRI Data Analysis

Preprocessing and data analysis were performed using SPM8 software (Wellcome Department of Imaging Neuroscience, London, UK). The functional images were temporally corrected for acquisition time differences with regard to the middle slice, realigned to the first image to correct for movement-related effects, coregistered to the anatomical image, and spatially smoothed with an isotropic Gaussian kernel (full width at half maximum = 8 mm). We conducted voxel-wise statistical analyses based on the general linear model. For the statistical model, an event-related design was modeled using the canonical hemodynamic response function and temporal derivative, and low-frequency drifts were removed using a high-pass filter (128 s). The onsets were defined as the onset time of the stimulus presented. We computed contrasts for “after > before (correct trial),” “after > before (incorrect trial),” “before > after (correct trial),” and “before > after (incorrect trial).” We assessed the statistical significance at a single-voxel threshold of *p* < 0.05, family-wise error (FWE)-corrected (voxel-level corrected), and activations that involved a contiguous cluster of at least 10 voxels were reported. MNI coordinates indicating the peak activation were converted to Talairach coordinates (47) using a non-linear transformation of the MNI brain image to the Talairach brain image (http://imaging.mrc-cbu.cam.ac.uk/imaging/MniTalairach). The active cortical areas were found using Talairach Client (48).

## Results

### Linguistic Assessments

The practice of MIT-J was started from Level I using words as stimuli. As the correct answer rate was over 90%, the training was moved to the next stage. By the ninth day of training, the patient had reached Level III using three syllable sentences (Figure S2 in Supplementary Material).

The results of the WAB and naming of 90 words are shown in Table 1. The spontaneous speech, repetition, and naming subscores of the WAB were all improved (higher) after the intervention. It is noteworthy that auditory comprehension also improved. There was a marked improvement in AQ, from moderate to above the cut-off between moderate and mild. The RT in the naming of 90 words task was also significantly shorter after MIT-J (*p* = 0.049, Wilcoxon signed-rank test).

**Table 1:** **Linguistic function before and after MIT-J**.

	Before	After
Western Aphasia Battery (WAB)		
Spontaneous speech	12	16
Auditory comprehension	7.95	8.85
Repetition	7.6	8.4
Naming	3.7	5
Reading	7.6	7.5
Writing	3.9	3.65
Aphasia quotient (AQ)	62.5	76.5
Naming of 90 words		
Correct	42	48
Response time (RT) (s)		
Mean	3.86	2.08
SE	0.74	0.35

*MIT-J, Japanese version of Melodic Intonation Therapy*.

### Changes in fMRI Activity Patterns

**Figure 2:**
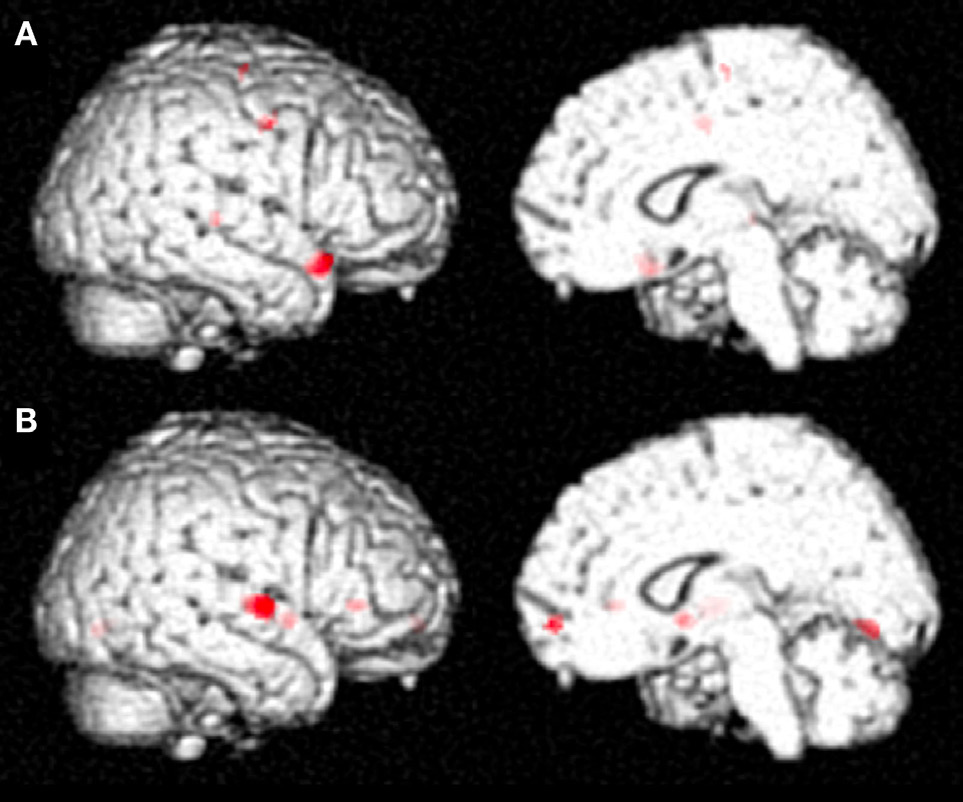
**(A)** Specific neural activation pattern on correct trials before MIT-J (Pre-MIT-J minus Post-MIT-J). There was substantial activation of the middle frontal gyrus, inferior frontal gyrus, the superior temporal gyrus, and precentral gyrus of the right hemisphere. **(B)** Specific neural activation pattern on incorrect trials after MIT-J (Post-MIT-J minus Pre-MIT-J) showing significant activation of the medial frontal gyrus, inferior frontal gyrus, superior temporal gyrus, lentiform nucleus, and lingual gyrus of the right hemisphere.

The differential activation pattern on correct trials before MIT-J minus after MIT-J (i.e., regions exclusively activated on correct trials before intervention) showed activation of the middle frontal gyrus, inferior frontal gyrus, superior temporal gyrus, and precentral gyrus of the right hemisphere (Figure 2; Table 2). In contrast, these regions showed no significant activation on incorrect trials before intervention. The differential activation pattern on incorrect trials after intervention minus before intervention (i.e., regions exclusively activated on incorrect trials after MIT-J) showed significant activation of the medial frontal gyrus, inferior frontal gyrus, superior temporal gyrus, lentiform nucleus, and lingual gyrus of the right hemisphere. In contrast, there was no significant activation of these regions on correct trials after MIT-J (post-MIT-J minus pre-MIT-J) (Figure 2; Table 2). Thus, the patient’s right hemisphere was relatively deactivated on correct naming trials after MIT-J.

**Table 2:** **Differential brain activation patterns before and after MIT-J**.

				Talairach coordinates (mm)				
Contrast	L/R	Area	Brodmann area	*X*	*Y*	*Z*	*Z* value	Cluster size in voxels
Before > after MIT (correct trials)	R	Inferior frontal gyrus	47	42	19	−16	6.57	79
	R	Superior temporal gyrus	22	48	−25	3	5.41	13
	R	Precentral gyrus	6	36	−6	37	5.28	36
	R	Precentral gyrus	6	46	−2	39	4.91	
	R	Middle frontal gyrus	6	26	−11	59	5.14	14
After > before MIT (incorrect trials)	R	Superior temporal gyrus	22	57	−8	2	6.93	128
	R	Lentiform nucleus		30	4	−2	6.53	56
	R	Medial frontal gyrus	10	6	56	−8	5.77	28
	R	Inferior frontal gyrus	47	36	29	0	5.74	26
	R	Lingual gyrus	18	14	−72	−1	5.19	43

## Discussion

The main findings of the present study are as follows: (i) MIT-J improved language output (as indicated by the spontaneous speech, repetition, and naming subscores of the WAB), consistent with results using the original version of MIT; (ii) auditory comprehension improved; (iii) the RT for figure naming was significantly shorter after MIT-J; (iv) the right hemisphere was relatively less activated on correct naming trials after MIT-J than on correct trials before intervention; and (v) the right hemisphere was strongly activated on incorrect trials after MIT-J compared with before. These results indicate that a relatively brief, but intensive, period of MIT-J can improve long-standing chronic aphasia, possibly by increasing neural processing efficiency.

Similar to results obtained using the original MIT, language production by a patient with chronic aphasia several years in duration was improved by MIT-J. It is notable that in addition to speak production, auditory comprehension was also improved. Although the phonological characteristics of English include melody, rhythm, and stress, those of Japanese include only melody and rhythm because of mora. Furthermore, four pitches are used in the original MIT, whereas MIT-J uses only two. Because of this simpler process, MIT-J may be applicable to a broader range of aphasia symptoms than the original MIT.

The improvement in figure naming implies that the number of words retrievable from memory increased after MIT-J, whereas the significant reduction in RT suggests that retrieval became faster. Thus, MIT-J appeared to have beneficial effects on both access and retrieval of words from memory. Our study is the first to demonstrate the efficacy of the RT of MIT by quantitative improvement.

Results of fMRI showed that the patient’s right hemisphere was relatively deactivated on correct trials after MIT-J. On the other hand, we also found substantial activation of the patient’s right hemisphere on incorrect trials after the intervention. It is generally believed that higher brain activity corresponds to enhanced cognitive function. However, brighter individuals displayed lower (more efficient) brain activation while performing cognitive tasks of lower difficult (49). Alternatively, individuals of greater intelligence often show greater activation during difficult tasks, corresponding to recruitment of additional resources. Such observations have given rise to neural efficiency theory (50) that posits that the efficient use of resources decreases cognitive load and thus enhances performance. Evidence for enhanced neural efficiency has also been reported in some aphasia studies (37, 51); Breier et al. (37) showed that a patient who responded positively to MIT exhibited decreasing activation within areas of the right-hemisphere homotopic to the left hemisphere language areas in the naming task, whereas Abel et al. (51) found that intensive lexical therapy for patients with chronic aphasia was associated with decreased cortical activation, attributed to higher processing efficiency within the naming network. In a similar way, brain activation in our patient was decreased compared with pre-intervention during the naming task because of curtailment of cognitive load. Thus, results demonstrate a link between MIT efficacy and neural efficiency by showing differences in activation patterns on correct versus incorrect trials. That is, relative deactivation on correct trials represents improved neural efficiency for effective naming, whereas the relatively higher activation on incorrect trials post-MIT-J represents the engagement of various neural resources for mastering the task.

In conclusion, brief, intensive MIT-J improved the language output of a chronic aphasia patient who had not responded to conventional speech therapy. Auditory comprehension was also improved. fMRI showed relative deactivation of the right hemisphere after the intervention, suggesting that MIT may enhanced neural efficiency, thereby decreasing cognitive load and improving language output.

## Author Contributions

KT and MS conceived and designed the experiments. KT, CN, and MS performed the experiments. KT, CN, and MS analyzed the data. KT and MS wrote the paper. AI, YS, and HK analyzed and the interpreted the data. HS and HT supervised and the interpreted the data.

## Conflict of Interest Statement

The authors declare that the research was conducted in the absence of any commercial or financial relationships that could be construed as a potential conflict of interest.

## Supplementary Material

Figure S1 **English (A) and Japanese (B) prosodic patterns and their transposition to melodic intonation patterns [from Seki and Sugishita (19)]**. MIT-J uses two pitches, high and low **(A)**, whereas original MIT uses several pitches **(B)**. H, high; L, low.

Figure S2 **Clinical course and MIT-J level of the present case**.
