# Supplementary figures and images for: Improved Neural Processing Efficiency in a Chronic Aphasia Patient Following Melodic Intonation Therapy: A Neuropsychological and Functional MRI Study
Source: Front Neurol. 2016 Sep 19;7:148. doi: 10.3389/fneur.2016.00148 (PMC5027199; doi:10.3389/fneur.2016.00148)

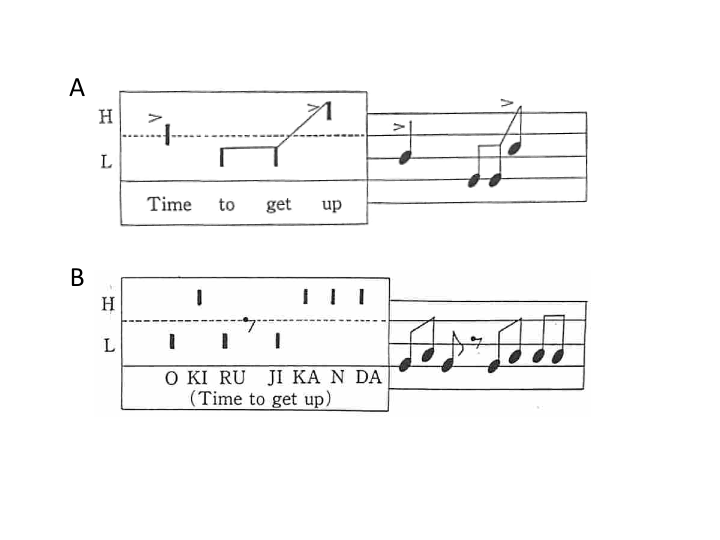

Supplement: Figure S1 — English (A) and Japanese (B) prosodic patterns and their transposition to melodic intonation patterns [from Seki and Sugishita (19)]. MIT-J uses two pitches, high and low (A), whereas original MIT uses several pitches (B). H, high; L, low. [file Image_1.TIFF]

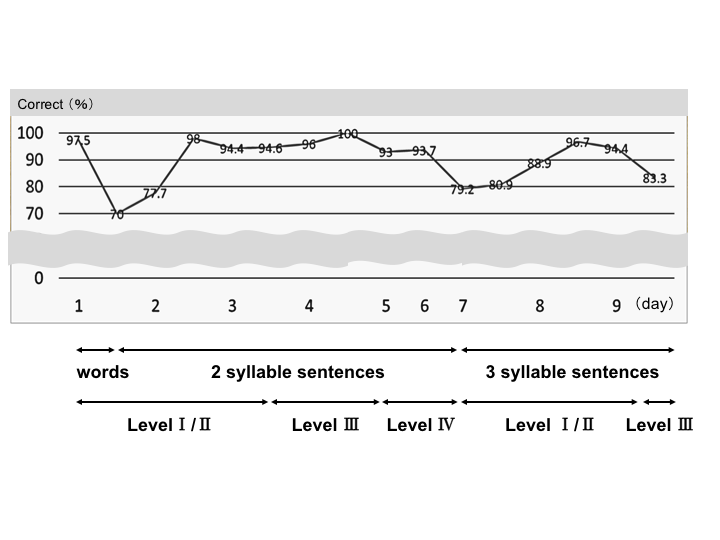

Supplement: Figure S2 — Clinical course and MIT-J level of the present case. [file Image_2.TIFF]
